# Predictors of B-line count in hospitalized patients with COVID-19

**DOI:** 10.3389/fcvm.2025.1618919

**Published:** 2025-11-21

**Authors:** Øyvind Johannessen, Caroline Espersen, Elke Platz, Kristoffer Grundtvig Skaarup, Mats Christian Højbjerg Lassen, Filip Soeskov Davidovski, Jacob Christensen, Jakob Øystein Simonsen, Anne Bjerg Nielsen, Alia Saed Alhakak, Niklas Dyrby Johansen, Morten Sengeløv, Kasper Iversen, Morten Schou, Peder L. Myhre, Tor Biering-Sørensen

**Affiliations:** 1 Institute for Clinical Medicine, University of Oslo, Oslo, Norway; 2 Department of Cardiology, Akershus University Hospital, Lørenskog, Norway; 3 Department of Cardiology, Herlev and Gentofte University Hospital, Copenhagen, Denmark; 4 Faculty of Health and Medical Sciences, University of Copenhagen, Copenhagen, Denmark; 5 Cardiovascular Division, Brigham and Women’s Hospital, Boston, MA, United States; 6 Harvard Medical School, Boston, MA, United States; 7 Department of Cardiology, Rigshospitalet, Copenhagen, Denmark; 8 Biomedical Sciences, Faculty of Health and Medical Sciences, University of Copenhagen, Copenhagen, Denmark; 9 Steno Diabetes Center Copenhagen, Copenhagen, Denmark

**Keywords:** lung ultrasound, cardiac troponins, cardiac biomarkers, echocardiography, COVID-19, B-lines

## Abstract

**Background:**

B-lines on lung ultrasound (LUS) are nonspecific signs of increased lung density, which can be secondary to pulmonary congestion, pneumonia, or fibrosis. While its use is recommended in acute heart failure (HF), its value in non-HF populations is less clear.

**Methods:**

We prospectively analyzed hospitalized non-ICU patients ≥18 years old with confirmed laboratory diagnoses of COVID-19. We aimed to identify associations between 8-zone LUS findings and clinical, laboratory, and echocardiographic data, and 30-day mortality using trend and regression analyses.

**Results:**

Among 270 patients (mean age 69 ± 14 years, 58% male, and 11% with prior HF) the median time from hospital admission to LUS was 4 days [interquartile range (IQR) 2–8]. In total, 263 (97%) had ≥1 B-line [median B-line number 13 (IQR 9–22)], and the median left ventricular ejection fraction (LVEF) was 59 (IQR 54–63). In adjusted models, having more B-lines was associated with higher levels of C-reactive protein, with an incidence rate ratio (IRR) of 16% (95%CI: 8%–24%) per log-unit increase (*P* < 0.001) and higher Early Warning Score [IRR 5% (95%CI: 2%–8%) per point, *P* = 0.003]. Higher tricuspid regurgitation gradient was associated with more B-lines: IRR 2% (95%CI: 1%–3%) per mmHg, *P* = 0.001). However, left ventricular function measures and NT-proBNP concentrations showed no significant association with B-lines. Furthermore, B-lines were not associated with 30-day mortality.

**Conclusions:**

Among patients with COVID-19, B-lines on LUS are associated with markers of infectious disease severity and pulmonary hypertension, but not with markers of left-sided HF.

## Introduction

The coronavirus disease 2019 (COVID-19) pandemic challenged healthcare systems worldwide as the severe acute respiratory syndrome coronavirus 2 (SARS-CoV-2) virus put patients at risk for respiratory complications and fatalities. The pandemic is no longer a public health emergency, shifting its management toward that of other common infectious diseases (1).

Lung ultrasonography (LUS) is a valuable tool for assessing patients with acute dyspnea and identifying those at risk for adverse outcomes. LUS can identify certain abnormalities in patients with pulmonary infections due to COVID-19 and these include vertical reverberation artifacts (B-lines), an irregular pleural line, and subpleural consolidations and correlate well with pulmonary parenchyma involvement, as seen on computerized tomography (CT) (2, 3). Given its feasibility and logistical advantages in isolated patients, LUS is recommended as a point-of-care tool when assessing patients with COVID-19 (4).

B-lines are not exclusive findings to COVID-19, and thickened alveolar septae and increased lung density are seen in conditions like heart failure (HF), bacterial pneumonia, and pulmonary fibrosis. In acute HF (AHF), B-lines are associated with pulmonary congestion, elevated filling pressures, and elevated natriuretic peptide levels. LUS is recommended during routine diagnostic workup of AHF (5–7).

Myocardial dysfunction and injury, demonstrated by echocardiographic abnormalities and cardiac-specific biomarkers, are common findings in patients with severe COVID-19 and are associated with disease severity and outcomes (8). While a recognized association exists between B-lines on LUS and congestion in AHF and between B-lines on LUS and COVID-19 severity, it is unknown what role myocardial dysfunction plays in the appearance of B-lines on LUS in patients with COVID-19.

This study aimed to examine the correlations between B-lines and clinical, echocardiographic, and laboratory findings in patients hospitalized during the first two waves of the COVID-19 pandemic. We also assessed the prognostic value of LUS regarding 30-day mortality.

## Methods

“*Echocardiography in COVID-19*” (ECHOVID-19) was a prospective, multicenter, observational study investigating the acute effect of COVID-19 on the heart and lungs by ultrasonography. The study specifically aimed to examine the role of ultrasonography in predicting short- and long-term cardiovascular outcomes. The ECHOVID-19 study enrolled 305 patients over 18 years of age with laboratory-confirmed SARS-CoV-2 during the first two waves of COVID-19 in Denmark. For inclusion, patients were required to have a positive polymerase chain reaction (PCR) test, age ≥18 years, and be admitted to non-ICU units. Patients unwilling or unable to understand the written informed consent were excluded. The study complied with the Declaration of Helsinki, and the regional ethics committee in the greater Copenhagen area approved the study. (Clinicaltrials.gov identifier NCT04377035).

### Lung ultrasound and echocardiography

Trained investigators performed the ultrasonographic examinations at the bedside using the portable Vivid IQ 4D ultrasound system (GE Healthcare, Horten, Norway) with a phased array transducer (1.7–3.3 MHz). Images were analyzed offline with commercially available software (EchoPAC version 203, GE, Vingmed Ultrasound AS). The echocardiographic images were analyzed by experienced investigators (ABN, FSD, JC), and one LUS investigator (CE) analyzed the LUS images offline, blinded to the clinical information, including clinical outcomes.

### Lung ultrasound imaging and analysis

The sonographers employed a pre-defined 8-zone LUS imaging protocol with the patients in a semi-recumbent position. The images were obtained with the transducer oriented sagittally at 18–20 cm imaging depth. An optimal image was defined as identifying pleural lung sliding between two ribs. Five- to six-second cine-loops were recorded in each lung zone (9). We excluded patients with ≥3 LUS zones missing based on prior consistent results with 6 zones analysis compared to 8 zones (10). Confluent B-lines were identified when ≥50% of the intercostal space was occupied by a continuous B-line and were counted as seven unique B-lines. In the absence of a standard B-line quantification approach in patients with COVID-19, we used three methods:

Method 1: The total number of B-lines in each LUS zone was summed across all eight zones. The maximum number of B-lines was 8 × 7 = 56 for patients with all zones available, 7 × 7 = 49 for patients with one missing zone, and 6 × 7 = 42 for patients with two missing zones.

Method 2: A LUS score ranging from 0 to 24 was constructed to include subpleural and lobar consolidations in addition to B-lines (9). For each lung zone, the presence of ≥3 B-lines was scored 1 point, the presence of confluent B-lines was scored 2 points and the presence of subpleural or lobar consolidations was scored 3 points. The LUS score was calculated by adding the score for all zones.

For Method 3, we explored a bilateral finding of ≥3 B-lines in ≥2 LUS zones as diagnostic for bilateral pneumonitis, according to current recommendations (11).

### Echocardiography

All patients underwent comprehensive echocardiography, simultaneously as LUS. Echocardiogram analyses followed a pre-specified protocol based on existing guidelines, and was previously published (12). Mitral valve peak early diastolic flow velocity (E-wave) was calculated relative to the average of lateral and septal wall tissue velocity (e’) to obtain the average E/e’ ratio, and the E-wave was indexed to late diastolic mitral inflow (A-wave) for the E/A ratio. The left atrial volume was adjusted to the body surface area to obtain the LAVi (12).

### Collection of clinical and laboratory data

All patients answered a questionnaire covering medical history, smoking, and medications. Information on body weight, height, and clinical parameters on the day of the ultrasound examination was retrieved through electronic health records. Routine laboratory measurements were conducted on the same day of ultrasound examinations or scheduled for the following day (9). N-terminal pro-B-type natriuretic peptide (NT-proBNP; Roche Diagnostics, Basel Switzerland), high-sensitive cardiac troponin I (hs-cTnI), and high-sensitive cardiac troponin T (hs-cTnT; Roche Diagnostics, Basel Switzerland) concentrations were sampled per research protocol when unavailable from the routine clinical lab, and clinically practical.to obtain. The presence of myocardial injury was defined as an elevated cardiac troponin (cTn) concentration above the 99th percentile at the respective centers, according to Danish standard practice. For cTnT, the cut-off concentration was 14 ng/L, and for cTnI, the cut-off concentrations was defined according to manufacturer and kit at the respective centers [45 ng/L [Gentofte and Herlev Hospital] and 59 ng/L [Hillerød, Roskilde, and Slagelse Hospital]].

All study sites used an early warning score (EWS) in their daily routine care (13). The EWS is a clinical monitoring and prognostication tool for identifying patients at risk for rapid deterioration. It comprises several vital parameters [heart rate, peripheral oxygen saturation (SpO2), respiration frequency, the requirement for supplementary oxygen, body temperature, and level of consciousness] into a single numeric value. The resulting EWS value provides nursing staff with triggers to prompt increased nursing attention, notify a physician, or alert an acute medical response team (14). The EWS is associated with disease severity and predicts in-hospital mortality, ICU admission, and outcomes, including those with COVID-19 (15).

### Outcome measures

The primary clinical outcome measure was 30-day all-cause mortality. Survival status was collected through electronic medical record review (9).

### Statistics

We present normally distributed continuous variables as means with standard deviation (SD), skewed variables as medians with interquartile ranges [IQR], and categorical variables as counts and (%). We applied appropriate tests according to the central tendency and data distribution.

Due to the lack of established clinically relevant cut-off values in the literature for B-lines in a COVID-19 cohort and a skewed B-line distribution with high variance, we analyzed baseline and outcome data based on three distinct approaches: (1) as tertiles of the total B-line number, (2) B-lines as a continuous variable and (3) a cut-off for the presence of ≥3 B-lines in ≥2 LUS zones.

In the first approach, each tertile represents increasing lung density, and was used previously to explore predictors of B-lines in HF (5). The total number of B-lines ranged from 0 to 46, with tertiles defined as follows: Tertile 1 (T1), 0–9 B-lines; Tertile 2 (T2), 10–16 B-lines; and Tertile 3 (T3) ≥ 17 B-lines. T2 had fewer patients due to B-line distribution (Supplementary Figure S1). Baseline clinical and demographic characteristics were stratified and tabulated according to B-line tertiles, and trends were assessed across the B-line tertiles using non-parametric trend tests and linear regression.

For the second approach, we used unadjusted and adjusted negative binomial (NB) regression models, with B-lines as the dependent variable, to assess their association with baseline characteristics, laboratory values, and echocardiographic parameters. We adjusted for variables previously associated with B-lines in LUS studies of AHF, healthy subjects, and COVID-19: Age, sex, BMI, CRP, and the wave of the pandemic (first vs. second wave) (16–19). The wave represents differences in time, differences in dominating virus strain (20C in the first wave vs. EU1 and Alpha B.1.1.7 in the second wave) (20), and differences in guideline-based therapy [systemic corticosteroids, antiviral agents (remdesivir) or both]. We report results from the NB regression models as ratios with 95% confidence intervals (CI) where a one unit change in the independent variable corresponds to a percentage increase or decrease in B-lines. Skewed variables [NT-proBNP, CRP, cTnT, cTnI, ferritin, BUN, hemoglobin, D-dimer, white blood cells (WBC), and lymphocytes] were log-transformed before regression analysis.

We used univariable and multivariable Cox proportional hazards regression models to estimate the hazard ratio (HR) with 95% CI for 30-day mortality in a time-to-event analysis from the time of imaging. Separate analyses were performed for the three analysis methods: (1) B-line tertiles, (2) B-line score, and (3) the presence of ≥3B-lines in ≥2 LUS zones. The multivariable models were only adjusted for age and sex, given the low number of events and the risk of overfitting the models.

To explore differences in baseline characteristics, laboratory values, echocardiographic parameters, and LUS findings according to cardiac injury and systemic inflammation, we categorized patients based on elevated troponin values and CRP levels above or below the median of 55 mmol/L.

A subset of patients with a prior history of HF was analyzed separately in a sensitivity analysis to assess the proportions of patients with B-lines above the median compared to those below the median number of B-lines.

Stata SE 17.0 (StataCorp, College Station, TX, USA), was used for all analyses, and a two-sided *P*-value of 0.05 was defined as statistically significant.

## Results

### Study population

**Figure 1:**
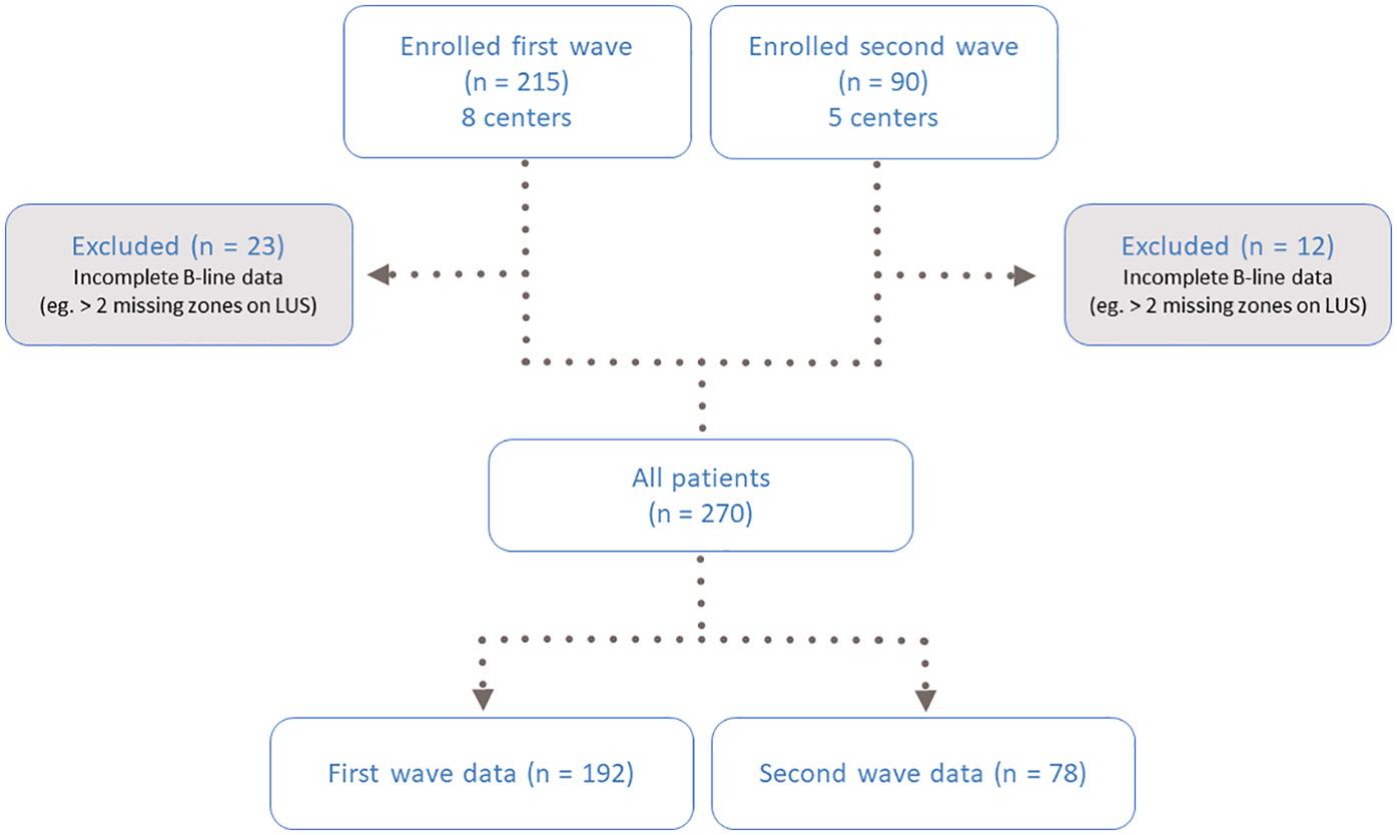
Flow chart of the study population. LUS, lung ultrasound.

In total, 215 patients were included from eight centers between March 30 and June 3, 2020 (first wave), and 90 patients from five centers between January 2021 and March 2021 (second wave). The cohort was previously described (20). Of the 305 patients included in the study, 35 were excluded due to missing data for ≥3 lung zones, leaving a final study population of 270 patients for this analysis (Figure 1). The mean age was 69 ± 14 years; 58% were male, 16% had chronic obstructive pulmonary disease (COPD), and 11% had a history of HF. The median time from hospital admission to LUS was 4 days (IQR 2–8).

The total number of B-lines across eight zones ranged from 0 to 46 with 7 (3%) patients having 0 B-lines. In the first wave, the median number of B-lines was 14 (IQR 9–22), which was higher than in the second wave [10 (IQR 4–15), *P* < 0.001] (Supplementary Table S1). LUS scores were also higher in the first wave [median 3[IQR 1–6] vs. 1[IQR 0–3], *P* < 0.001]. Similarly, a bilateral finding of ≥3 B-lines in ≥2 LUS zones, considered the LUS equivalent of bilateral pneumonitis, was more frequent in the first vs. second wave [44 [23%] vs. 5 [6%], respectively, *P* = 0.001].

### Association of lung ultrasound with clinical characteristics

**Figure 2:**
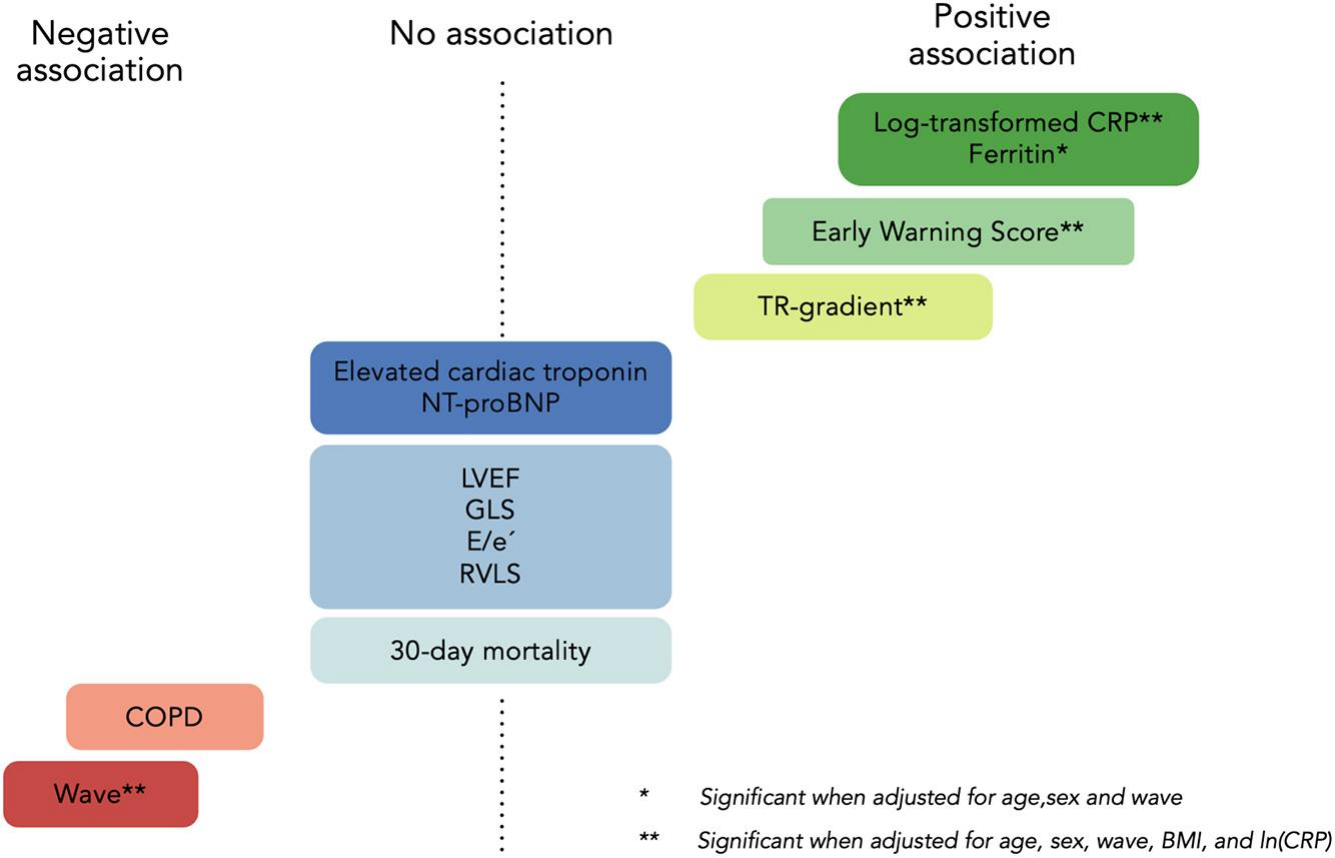
Predictors of B-lines in COVID-19. IRR, incident rate ratio. *Significant in adjusted model 1 (age, sex, and wave). **Retained significance after correcting for age, sex, wave, BMI, and log-transformed CRP (model 2). Wave reflects different virus strains ((20C in the first wave vs. EU1 and Alpha B.1.1.7 in the second wave) and treatment with systemic corticosteroids and antiviral medication. COPD, chronic obstructive pulmonary disease, E/e'=early diastolic mitral inflow relative to cardiac tissue velocity, GLS, global longitudinal strain; LVEF, left ventricular ejection fraction; NT-proBNP, N-terminal pro B-type natriuretic peptide; RVLS, right ventricular longitudinal strain; TR, tricuspid regurgitation. Reproduced with permisison from “Predictors of B-lines” by O Johannessen, C Espersen, E Platz, K G Skaarup, M C H Lassen, F S Davidovski, J Christensen, JØ Simonsen, A B Nielsen, N D Johansen, M Sengeloev, K K Iversen, M Schou, P L Myhre and T B Sorensen. European Heart Journal, Oxford University Press.

There were no significant differences in age, sex, BMI, and smoking status across tertiles of B-lines. A trend toward fewer B-lines in patients with COPD (*P* = 0.02) was attenuated in the adjusted regression analyses (Figure 2; Tables 1 and 2). Other comorbidities, including HF, were not associated with a higher number of B-lines (Table 1).

**Table 1:** Baseline clinical, laboratory, and echocardiographic characteristics according to B-line tertiles.

Variables	*N*	Total	Tertile 1	Tertile 2	Tertile 3	*P* trend
			0–9 B-lines	10–16 B-lines	≥17 B-lines	
		*N* = 270	*N* = 94	*N* = 85	*N* = 91	
Clinical characteristics						
Age, years	270	69 (14)	69 (15)	70 (15)	68 (13)	0.75
Male, *n* (%)	270	156 (58%)	49 (52%)	49 (58%)	58 (64%)	0.11
BMI, kg/m^2^	252	25 (23–29)	25 (23–27)	26 (23–31)	25 (23–29)	0.55
Smoking (active), *n* (%)	245	16 (7%)	5 (6%)	4 (5%)	7 (9%)	0.51
Hypertension, *n* (%)	270	136 (50%)	46 (49%)	47 (55%)	43 (47%)	0.83
Diabetes, *n* (%)	269	56 (21%)	20 (22%)	18 (21%)	18 (20%)	0.79
Prevalent heart failure, *n* (%)	270	30 (11%)	8 (9%)	12 (14%)	10 (11%)	0.59
Previous ischemic heart disease, *n* (%)	268	29 (11%)	10 (11%)	8 (10%)	11 (12%)	0.77
Atrial fibrillation/flutter, *n* (%)	270	55 (20%)	15 (16%)	17 (20%)	23 (25%)	0.12
COPD, *n* (%)	270	43 (16%)	22 (23%)	11 (13%)	10 (11%)	0.021
Asthma, *n* (%)	269	36 (13%)	16 (17%)	9 (11%)	11 (12%)	0.32
Other lung disease^a^, *n* (%)	263	17 (6%)	4 (4%)	8 (10%)	5 (6%)	0.73
Vital signs						
Systolic blood pressure, mmHg	270	127 (19)	127 (20)	132 (20)	123 (16)	0.15
Heart rate, BPM	270	80 (17)	81 (17)	77 (17)	84 (15)	0.27
Temperature, Celsius	270	37.0 (0.7)	37.0 (0.7)	37.0 (0.7)	37.1 (0.6)	0.22
Respiratory rate, per/min	270	18 (17–20)	18 (16–20)	19 (17–20)	20 (18–21)	<0.001
Oxygen saturation, %	270	95 (93–97)	95 (94–97)	95 (94–97)	94 (93–96)	0.013
Oxygen therapy, L/min	266	0.8 (0.0–2.5)	0.0 (0.0–1.0)	0.0 (0.0–2.0)	2.0 (0.5–5.0)	<0.001
EWS score, points	270	3 (1–5)	2 (1–4)	3 (1–4)	4 (2–6)	<0.001
Biochemistry						
Blood pH	204	7.46 (0.05)	7.46 (0.06)	7.46 (0.04)	7.47 (0.05)	0.23
PCO_2_, kPa	202	4.7 (4.2–5.3)	4.7 (4.3–5.1)	4.8 (4.2–5.3)	4.7 (4.2–5.2)	0.50
Lactate, mmol/L	172	1.2 (0.9–1.7)	1.4 (1.0–2.1)	1.1 (0.8–1.6)	1.3 (0.9–1.6)	0.25
Bicarbonate, mmol/L	195	26 (3)	26 (3)	26 (3)	26 (3)	0.98
WBC, ×10^9^/L	264	7.0 (5.0–9.9)	6.7 (4.7–9.4)	7.2 (5.2–10.4)	7.7 (5.2–10.0)	0.30
Lymphocytes, ×10^9^/L	262	1.1 (0.7–1.5)	1.2 (0.8–1.5)	1.1 (0.8–1.5)	1.1 (0.6–1.5)	0.07
C-reactive protein, mg/L	261	55 (23–93)	37 (12–75)	55 (22–89)	67 (34–133)	<0.001
Ferritin, µg/L	209	605 (301–1,240)	384 (201–856)	653 (338–1,450)	770 (465–1,280)	<0.001
Procalcitonin, µg/L	89	0.2 (0.1–0.5)	0.2 (0.1–0.4)	0.2 (0.1–0.6)	0.2 (0.1–0.6)	0.51
Creatinine, µmol/L	267	75 (58–100)	76 (57–92)	78 (60–103)	72 (59–97)	0.70
Blood urea nitrogen, mmol/L	252	5.9 (4.0–9.5)	5.8 (4.3–9.5)	5.5 (3.8–10.7)	6.2 (4.0–8.9)	0.78
Hemoglobin, mmol/L	267	7.4 (1.1)	7.5 (1.2)	7.5 (1.1)	7.2 (1.1)	0.11
D-dimer, mg/L (FEU)	179	1.3 (0.7–2.3)	1.1 (0.5–2.3)	1.2 (0.7–2.2)	1.5 (0.9–2.5)	0.07
NT-proBNP, ng/L	169	408 (178–1,522)	397 (171–1,260)	664 (211–1,810)	384 (118–1,581)	0.83
Elevated troponin, *n* (%)^b^	179	82 (46%)	25 (42%)	30 (53%)	27 (43%)	0.98
Echocardiography						
LVEF, %	198	59 (54–63)	59 (54–63)	58 (54–63)	59 (51–62)	0.29
LVMi, g/m^2^	188	84.5 (26.7)	84.4 (25.2)	86.2 (29.5)	83.2 (26.1)	0.79
GLS, %	240	−15.8 (4.4)	−15.9 (4.3)	−15.7 (4.5)	−15.8 (4.3)	0.90
LAVi, mL/m^2^	197	20.2 (15.2–26.1)	19.3 (14.8–24.7)	22.2 (16.4–30.1)	19.6 (14.9–26.1)	0.82
E/A	218	1.0 (0.8–1.2)	1.0 (0.8–1.2)	1.0 (0.8–1.3)	1.1 (0.9–1.4)	0.14
E/e’ (average)	165	8 (7–12)	8 (7–12)	9 (7–12)	8 (7–11)	0.94
TAPSE, cm	218	2.0 (0.5)	2.0 (0.5)	2.1 (0.4)	2.0 (0.5)	0.22
Peak TR gradient, mmHg^b^	164	23 (10)	19 (10)	22 (9)	25 (9)	0.002
TRV max >2.8 m/s *n* (%)	164	31 (18.9)	5 (11.6)	9 (17.6%)	17 (24.3%)	0.09
TRV max m/s	164	2.3 (0.6)	2.1 (0.6)	2.3 (0.5)	2.4 (0.5)	0.002
RV free wall longitudinal strain, (%)	138	−18.8 (−24.4 to −15.6)	−21.1 (−26.8 to −15.5)	−17.5 (−22.2 to −15.8)	−18.8 (−23.7 to −15.4)	0.30
LUS						
Total number of B-lines, *n*	270	13 (8–19)	6 (3–8)	13 (11–15)	22 (19–28)	<0.001
LUS score, points	270	2 (1–5)	0 (0–1)	2 (2–3)	6 (5–8)	<0.001
Bilateral pneumonitis, *n* (%)	270	49 (18%)	0 (0%)	2 (2%)	47 (52%)	<0.001

BMI, body mass index; BPM, beats per minute; COPD, chronic obstructive pulmonary disease; E/A, early diastolic mitral inflow relative to late diastolic mitral inflow; E/e’, early diastolic mitral inflow relative to cardiac tissue velocity; EWS, early warning score; FEU, fibrinogen equivalent units; GLS, global longitudinal strain; kPa, kilopascals; LAVi, left atrial volume index; LVEF, left ventricular ejection fraction; LVMi, left ventricular mass index; LUS, lung ultrasound; NT-proBNP, N-terminal pro B-type natriuretic peptide; PCO2, partial pressure of carbon dioxide; RV, right ventricular; TAPSE, tricuspid annular plane systolic excursion; TR, tricuspid regurgitation; WBC, white blood cells. Continuous variables are presented as mean (SD) or median (IQR). Proportions are presented as N (%).

a Sarcoidosis, lung fibrosis, etc.

b Cardiac troponin I and cardiac troponin T above their cut-off for the 99th percentile according to the respective centers.

**Table 2:** Univariable and multivariable negative binomial regression analysis assessing parameters associated with B-line count.

Variables	Unadjusted		Adjusted model 1		Adjusted model 2	
	IRR (95% CI)	*P*	IRR (95% CI)	*P*	IRR (95% CI)	*P*
Clinical characteristics						
Age, (per 5 years)	0.99 (0.96–1.02)	0.60	0.99 (0.97–1.02)	0.77	0.99 (0.97–1.02)	0.69
Male	1.13 (0.97–1.33)	0.13	1.14 (0.98–1.33)	0.08	1.08 (0.93–1.26)	0.33
BMI, kg/m^2^	0.997 (0.98–1.01)	0.73	1.00 (0.99–1.01)	0.96	0.99 (0.98–1.01)	0.75
Prevalent heart failure	1.11 (0.87–1.43)	0.40	1.14 (0.89–1.46)	0.29	1.13 (0.89–1.42)	0.32
COPD	0.79 (0.64–0.98)	0.03	0.82 (0.66–1.01)	0.07	0.84 (0.68–1.02)	0.08
Wave (2nd vs. 1st wave)	0.63 (0.53–0.75)	<0.001	0.63 (0.53–0.74)	<0.001	0.68 (0.58–0.80)	<0.001
Vital signs						
Systolic blood pressure, (per 5 mmHg)	0.98 (0.96–0.998)	0.03	0.98 (0.96–1.00)	0.11	0.995 (0.97–1.02)	0.66
Heart rate, BPM	1.00 (0.9996–1.01)	0.07	1.00 (1.00–1.01)	0.03	1.00 (0.999–1.01)	0.11
Temperature, Celsius	1.06 (0.94–1.19)	0.34	0.99 (0.88–1.11)	0.82	0.95 (0.85–1.06)	0.38
Respiratory rate, per min	1.03 (1.01–1.05)	0.006	1.03 (1.01–1.04)	0.007	1.02 (0.99–1.04)	0.06
Oxygen saturation, %	0.97 (0.94–1.00)	0.05	0.96 (0.93–0.99)	0.009	0.96 (0.93–0.99)	0.01
Oxygen therapy, L/min	1.05 (1.03–1.07)	<0.001	1.05 (1.03–1.07)	<0.001	1.04 (1.02–1.06)	<0.001
EWS score, points	1.07 (1.03–1.10)	<0.001	1.07 (1.03–1.10)	<0.001	1.05 (1.02–1.08)	0.003
Biochemistry						
Blood pH	1.95 (0.33–11.5)	0.46	1.50 (0.27–8.3)	0.64	1.44 (0.28–7.49)	0.67
Ln-Lactate, mmol/L	0.95 (0.78–1.15)	0.57	0.98 (0.82–1.18)	0.85	0.997 (0.83–1.19)	0.97
Ln-WBC, ×10^9^/L	1.08 (0.92–1.27)	0.34	1.21 (1.03–1.42)	0.02	1.21 (1.03–1.42)	0.02
Ln-C-reactive protein, mg/L	1.19 (1.11–1.27)	<0.001	1.16 (1.09–1.25)	<0.001	1.16 (1.08–1.24)	<0.001
Ln-Ferritin, µg/L	1.15 (1.06–1.25)	0.001	1.13 (1.04–1.23)	0.003	1.07 (0.99–1.17)	0.10
Ln-Procalcitonin, µg/L	1.00 (0.91–1.10)	0.98	1.01 (0.92–1.11)	0.84	0.99 (0.91–1.10)	0.98
Ln-Creatinine, µmol/L	1.01 (0.87–1.16)	0.91	0.98 (0.85–1.14)	0.81	0.96 (0.83–1.11)	0.62
Hemoglobin, mmol/L	0.94 (0.88–1.01)	0.09	0.94 (0.88–1.01)	0.10	0.96 (0.89–1.02)	0.17
Ln-D-dimer, mg/L (FEU)	1.06 (0.96–1.17)	0.27	1.05 (0.96–1.16)	0.28	1.02 (0.93–1.12)	0.66
Ln-NT-proBNP, (ng/L)	0.99 (0.93–1.05)	0.66	1.04 (0.96–1.11)	0.33	1.02 (0.95–1.09)	0.67
Elevated troponins, *n* (%)^a^	1.00 (0.83–1.22)	0.96	0.95 (0.78–1.17)	0.64	0.93 (0.76–1.14)	0.48
Echocardiography						
LVEF, %	0.99 (0.98–1.00)	0.23	0.99 (0.97–0.997)	0.01	0.998 (0.98–0.99)	0.02
LVMi, g/m^2^	0.999 (0.996–1.00)	0.62	1.00 (0.997–1.00)	0.70	1.00 (0.997–1.00)	0.55
GLS, %	1.00 (0.98–1.02)	0.99	1.01 (0.99–1.03)	0.19	1.02 (0.99–1.04)	0.07
LAVi, mL/m^2^	1.00 (0.997–1.01)	0.33	1.01 (1.00–1.02)	0.01	1.01 (1.00–1.02)	0.006
E/A	1.05 (0.99–1.11)	0.08	1.04 (0.98–1.09)	0.22	1.04 (0.99–1.10)	0.15
E/e’ (average)	0.99 (0.98–1.01)	0.62	1.00 (0.98–1.02)	0.80	1.00 (0.99–1.02)	0.79
TAPSE, cm	0.90 (0.75–1.07)	0.22	0.92 (0.77–1.09)	0.33	0.91 (0.77–1.08)	0.28
TR gradient, mmHg^b^	1.01 (1.00–1.02)	0.007	1.02 (1.01–1.03)	0.001	1.02 (1.01–1.03)	0.001
RV free wall longitudinal strain, %	1.01 (0.99–1.03)	0.41	1.01 (0.99–1.03)	0.40	1.01 (0.99–1.03)	0.31

BMI, body mass index; BPM, beats per minute; COPD, chronic obstructive pulmonary disease; E/A, early diastolic mitral inflow relative to late diastolic mitral inflow; E/e’, early diastolic mitral inflow relative to cardiac tissue velocity; EWS, early warning score; FEU, fibrinogen equivalent units; GLS, global longitudinal strain; LAVi, left atrial volume index; LVEF, left ventricular ejection fraction; LVMi, left ventricular mass index; LUS, lung ultrasound; NT-proBNP, N-terminal pro B-type natriuretic peptide; RV, right ventricular; TAPSE, tricuspid annular plane systolic excursion; TR, tricuspid regurgitation; WBC, white blood cells. Skewed laboratory variables are ln-transformed. Model 1 is adjusted for age, sex and wave. Model 2 is adjusted for age, sex, wave, BMI, and ln (CRP).

a Cardiac troponin I and cardiac troponin T above their cut-off for the 99th percentile according to the respective centers.

b In the 164 patients with a measurable peak tricuspid regurgitation continuous wave Doppler signal.

Patients with more B-lines had higher respiratory rates, lower peripheral oxygen saturation, and greater need for supplemental oxygen, which translates to a higher EWS (*P* < 0.001). In adjusted regression analyses, we found a 5% increase in B-lines for each point increase in EWS (95% CI: 2%–8%, *P* = 0.003) (Figure 2 and Table 2).

### Lung ultrasound’s association with circulating biomarkers

Patients with more B-lines had higher concentrations of CRP and ferritin. CRP was positively associated with a 19% increase in B-lines (95% CI: 11%–27%, *P* < 0.001) for each log-unit increase in CRP. This association persisted in the adjusted model showing a 16% increase in B-lines per log-unit increase in CRP (95% CI: 8%–24%, *P* < 0.001). There were no associations between B-lines and procalcitonin, creatinine, blood urea nitrogen (BUN), hemoglobin, WBC, or arterial blood gas analysis. D-dimer levels were elevated overall in the cohort [median 1.3 mg/L, (IQR 0.7–2.3)] with a non-significant trend across B-line tertiles (*P* = 0.07).

In total, 179 (66%) patients had available measurements of cTn. cTnI was measured in 86 patients [median 13 ng/L (IQR 7.7–36)], and cTnT was measured in 93 patients [median 22 ng/L (IQR 13–36)]. Eighty-two (46%) patients had cTn levels above the 99th percentile. However, no association was found between the number of B-lines and cTn values above or below the 99th percentile. (Table 1 and Supplementary Table S2).

### Lung ultrasound’s association with cardiac structure and function

LV systolic function (LVEF and GLS), LV diastolic function (E/e’ ratio and LAVi), and RV function (TAPSE and RV strain) were not significantly associated with the number of B-lines in trend or regression analysis. There was a statistically significant association between B-lines tertiles and peak TR-gradient (*P* = 0.002, available in *n* = 164) (Figure 2, Table 1). The association between TR-gradient and B-lines persisted in univariable and multivariable regression models adjusting for age, sex, BMI, wave, and CRP [2% increase in B-lines per 1 mmHg increase in TR-gradient (95% CI: 1%–3%), *P* = 0.001] (Table 2).

### B-lines in patients with known heart failure

In the subgroup of patients with a history of HF (*n* = 30, 11%), those with B-lines above the median (≥14 B-lines) had similar measurements of LVEF, GLS, E/e’, NT-proBNP and cTn compared to patients with <14 B-lines (Supplementary Table S3).

### Lung ultrasound and clinical variables according to systemic inflammation

Patients with severe systemic inflammation (CRP above median, ≥55 mg/L) had more B-lines and a higher LUS score compared to patients with CRP below the median [median B-lines of 16 vs. 12, [*P* = 0.001], and median LUS score of 3 vs. 2, [*P* = 0.006], respectively]. Patients with CRP levels above the median were older, more likely to be male, and had more severe vital sign abnormalities (higher EWS) than those with CRP levels below the median. They also had values corresponding to a more severe inflammatory state, with higher cTnI levels (18 ng/L vs. 9 ng/L, respectively, *P* = 0.002); however, there were no statistically significant differences in echocardiographic variables (Supplementary Table S4).

### Association between LUS findings and clinical outcomes

**Figure 3:**
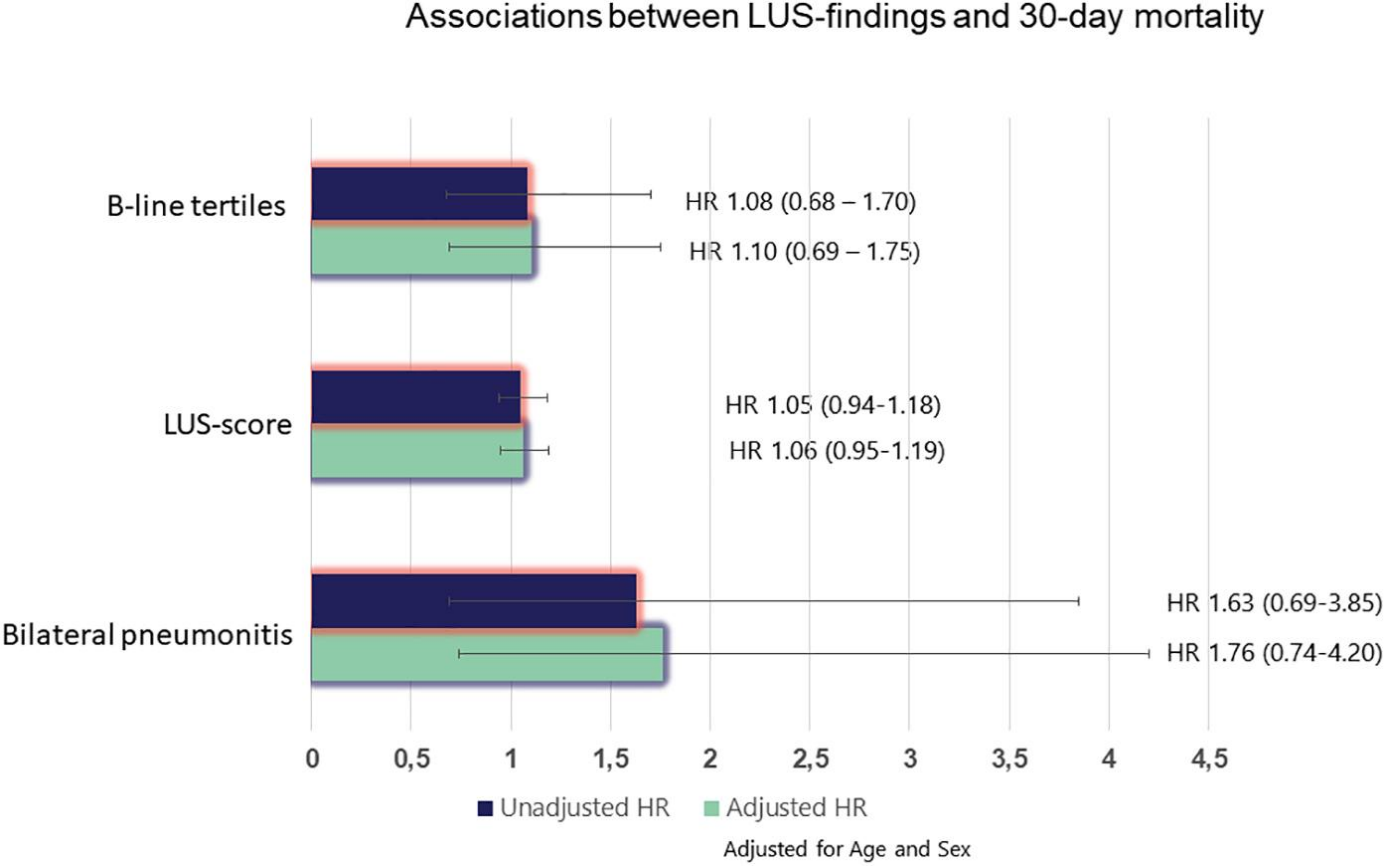
Associations between LUS findings and 30-day mortality. *Adjusted for age and sex. HR, hazard ratio.

During the 30-day follow-up after enrollment, there were 36 deaths. Neither the B-line tertiles (HR 1.08, 95% CI: 0.68–1.70), the LUS score [HR 1.05, 95% CI: 0.94–1.18)] nor bilateral pneumonitis (HR 1.63, 95% CI: 0.69–3.85) were associated with 30-day mortality in adjusted or unadjusted analysis (Figure 3).

## Discussion

This multisite prospective study assessing clinical, echocardiographic and laboratory variables associated with B-lines on LUS in patients with COVID-19 has three main findings. First, there were no associations between B-lines and abnormal cardiac function and structure. Second, B-lines strongly correlated with abnormal vital signs, disease severity markers, and inflammation, as well as the different pandemic waves. Third, LUS findings were not associated with 30-day mortality. These results highlight the utility of LUS when assessing clinical severity and pulmonary involvement in COVID-19, but also clarify the limited role for B-lines as indicators of cardiac dysfunction or mortality.

LUS is increasingly used for patient management in acute dyspnea (5), and, the European Society of Cardiology guidelines recommend LUS in the diagnostic work-up, during hospitalization, and before discharge in patients hospitalized with AHF (6). Multiple studies demonstrate LUS’s utility in detecting and quantifying pulmonary congestion, predicting short- and long-term adverse events, and providing treatment guidance in AHF (5, 22). LUS studies in COVID-19 demonstrate more inconsistent results. While most studies on COVID-19 report an abundance of LUS findings, such as B-lines and sub-pleural consolidations, the performance in risk stratification seems less effective than in patients with HF (23).

A multicenter study from the first COVID-19 wave in Eastern Denmark found that LUS predicted a higher risk of thromboembolic events but considered a suboptimal tool for identifying patients at risk of acute respiratory distress, need for mechanical ventilation, or in-hospital mortality (9). However, the majority of studies in COVID-19 have identified associations between LUS and worse outcomes (24). We also noted a significant reduction in LUS findings between the first and second waves, mirroring results from a prior study comparing LUS findings between the wild-type and Omicron COVID-19 variants, consistent with decreased pulmonary involvement between different waves (19). Our contradictory findings could be attributed to milder clinical presentation in the second wave alongside improved therapeutic strategies and vaccines contributing to improved outcomes and fewer events over time.

### Pathophysiologic explanations of B-lines

B-lines on LUS are vertical reverberation artifacts associated with ground glass opacities seen on chest CT. Ground glass opacities are non-specific radiological markers of abnormalities in the pulmonary interstitial tissue and typically represent cardiogenic pulmonary edema in patients with HF. Numerous studies have found strong correlations between B-lines and indicators of elevated intracardiac pressures, such as pulmonary capillary wedge pressure, NT-proBNP, the E/e’ ratio, and clinical congestion scores (7, 25). In patients hospitalized with COVID-19, B-lines are related to pulmonary inflammation, as shown in the current and similar studies examining LUS in pulmonary diseases, suggesting inflammation or infectious processes (26).

Fewer B-lines in patients with COPD than in those without COPD may be explained by a lower threshold for admitting patients with COPD due to their increased risk of complications rather than the severity of their COVID-19 infection. Another explanation may be that by the time of LUS, COPD patients had already been treated with systemic corticosteroids, which was standard treatment during the second wave, reducing pulmonary inflammation and B-lines.

Patients with higher peak TR-gradients, indicative of higher pulmonary artery pressures (PAP), had more B-lines. However, the peak TR gradients remained within the normal range, suggesting B-lines may be sensitive to subtle changes in cardiac function before other echocardiographic measures show notable impairment. The peak TR-gradient is likely elevated due to pulmonary stress caused by the infection and hypoxia. Other studies on COVD-19 have demonstrated elevated PAP below pathological levels in patients with persistent symptoms recovering from COVID-19 and one study found a relationship between B-lines and increased PAP in COVID (27, 28).

Another study comparing RV changes in bacterial pneumonia vs. COVID-19 infection found a significantly elevated peak TR-gradient in both diseases, albeit with milder changes for COVID-19 (29). Similarly, RV changes have been demonstrated in previous influenza pandemics, supporting pulmonary infection as the driver for the increased PAPs. Increased PAP seems to be a greater predictor for B-lines rather than measures of intracardiac pressures and stress, reflected by E/e’ ratio and NT-proBNP. Whether combining PAP and B-lines provide a more accurate assessment of disease progression and recovery in COVID-19 is interesting to explore in patients with persistent symptoms after the initial infection (28).

### The interaction between B-lines, heart failure, and pneumonia

Interestingly, in our subgroup analysis of patients with a history of prior HF, the number of B-lines was not associated with markers of cardiac filling pressures, (LAVi, E/e’ ratio, and NT-proBNP). The small size of this subgroup, however, limits definitive conclusions about this lack of a correlation, as larger pre-COVID studies of concomitant pneumonia in AHF found that B-line assessment alone is sufficient for diagnosing HF-related pulmonary congestion regardless of concomitant pneumonia (30). Nonetheless, this underscores the importance of considering differential diagnoses for B-lines in dyspneic patients, including those with prior HF. Specifically, if there is a concern for a concomitant pulmonary infection of any kind, integrating B-lines with findings of subpleural and pleural thickening could improve diagnostic accuracy as these are not typically present in cardiogenic pulmonary edema.

None of our quantification methods for B-lines were predictive of 30-day mortality, most likely due to the low number of events. Comparing the CI for the three different quantification methods suggests that incorporating additional pathological findings to B-lines and/or bilateral pneumonitis could provide more precise prognostic information. This aligns with results from studies using baseline LUS scores for predicting mortality or worsening of disease (31). Furthermore, in patients with HF, B-lines tend to predict future HF events, (i.e.,HF hospitalizations) but rarely mortality (21). Since we did not collect data on HF hospitalizations in this study, the usefulness of LUS in predicting HF-related occurrences in a COVID population remains unclear.

### Strengths and limitations

The following enhance the external generalizability of our study: (1) The prospective multicenter design; (2) echocardiographic and cardiac biomarkers were sampled specifically for this study, reducing selection bias towards patients with cardiac comorbidities; (3) the results were independent of the two waves and (4) a dedicated team of sonographers, blinded to baseline characteristics and outcomes, with similar training used a standardized imaging protocol with the same equipment.

Several limitations should be noted. The advantages obtained with a dedicated team come at the cost of delayed imaging. The time from hospitalization to LUS and echocardiography was four days. The optimal time-to-LUS is uncertain, but patients hospitalized with AHF demonstrate rapid B-line reduction during the first few hours after initiating treatment, with further decline within the first 48 h (32). Patients with known HF may have had more LUS findings during the first few hours and days of hospitalization and LUS artifacts related to congestion may have been less pronounced at the time of ultrasonography. The residual B-lines at imaging may therefore primarily be associated with the pulmonary infection. A second limitation is that the LUS imaging protocol did not include posterior thoracic zones where subpleural and lobar consolidations are often depicted and reported to correlate with CT findings in COVID-19 (33). The posterior zones are generally less accessible in respiratory distressed patients and it is reasonable to assume that adding posterior zones to the lateral and frontal zones, might capture data missed by our 8-zone protocol.

Lastly, the study was terminated early due to declining hospitalizations for COVID-19, limiting the ability to detect relevant associations with outcomes.

### Clinical application of the study

Measuring B-lines may be useful for risk-stratifying patients with COVID-19. A higher number of B-lines is primarily associated with greater severity of the pneumonia, as reflected in more inflammation, hypoxia, and elevated PAP. Clinicians should be aware that this is different from AHF, where B-lines are mostly associated with increased intracardiac pressure and pulmonary congestion.

## Conclusions

LUS findings were common among patients hospitalized with SARS-CoV-2 during the initial two COVID waves. The interpretation of LUS findings in HF should be considered in the context of ongoing pulmonary infection.

## Data Availability

The datasets presented in this article are not readily available due to legal reasons since personal information is included. Further enquiries can be directed toward the corresponding author.
