# Supplementary material for: Predictors of B-line count in hospitalized patients with COVID-19
Source: Front Cardiovasc Med. 2025 Nov 21;12:1618919. doi: 10.3389/fcvm.2025.1618919 (PMC12679293; doi:10.3389/fcvm.2025.1618919)
Supplement: Supplementary file 1 [file Datasheet1.docx]

Supplemental material

**Figure S1. Distribution of Total number of B-lines**

**
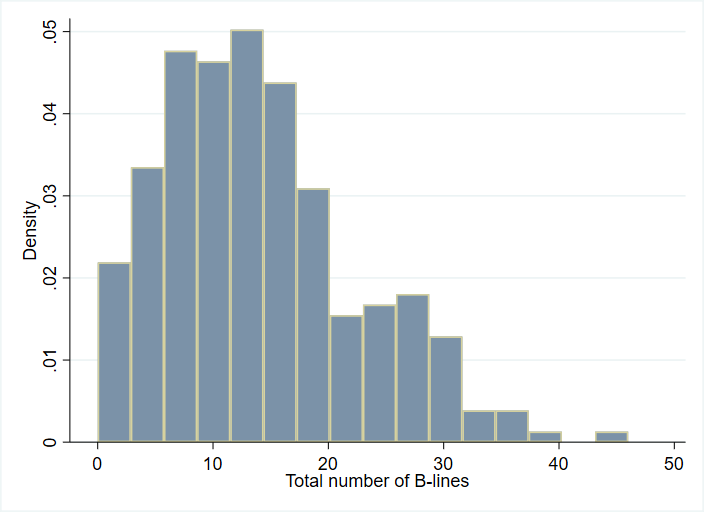
**

**Supplemental table S1. Differences in LUS findings from 1^st^ to 2^nd^ Wave.**

|  | **1^st^ Wave** | **2^nd^ Wave** | **P** |
| --- | --- | --- | --- |
| Total number of B-lines | 14 [9, 22] | 10 [4, 15] | <0.001 |
| LUS score | 3 [1, 6] | 1 [0, 3] | <0.001 |
| Bilateral pneumonitis, n (%) * | 44 (23%) | 5 (6%) | 0.001 |

*Bilateral pneumonitis =≥3 B-lines in ≥ thoracic zones bilaterally

**Supplemental table S2. Clinical, echocardiographic and lung ultrasound findings according to elevated Troponin I or T (n=179).**

| **Variables** | **Total** | **Non-elevated TnI/TnT** | **Elevated TnI/TnT** | **P** |
| --- | --- | --- | --- | --- |
|  | N=179 | N=97 | N=82 |  |
| **Clinical characteristics** |  |  |  |  |
| Age, years | 70 (14) | 66 (14) | 75 (12) | <0.001 |
| Male, n (%) | 104 (58%) | 53 (55%) | 51 (62%) | 0.31 |
| BMI, kg/m^2^ | 25 (22-28) | 25 (23-27) | 25 (22-29) | 0.84 |
| Active smoking, n (%) | 13 (8%) | 6 (7%) | 7 (9%) | 0.57 |
| Pack years | 20 (10-30) | 20 (8-30) | 25 (12-45) | 0.26 |
| **Medical history** |  |  |  |  |
| Hypertension, n (%) | 97 (54%) | 44 (45%) | 53 (65%) | 0.010 |
| Diabetes, n (%) | 41 (23%) | 21 (22%) | 20 (25%) | 0.63 |
| Prevalent heart failure, n (%) | 21 (12%) | 3 (3%) | 18 (22%) | <0.001 |
| Previous ischemic heart disease, n (%) |  |  |  |  |
| Atrial arrythmia | 21 (12%) | 6 (6%) | 15 (19%) | 0.02 |
| COPD, n (%) | 29 (16%) | 11 (11%) | 18 (22%) | 0.06 |
| Asthma, n (%) | 22 (12%) | 13 (13%) | 9 (11%) | 0.82 |
| Other lung disease*, n(%) | 12 (7%) | 4 (4%) | 8 (10%) | 0.15 |
| **Vital signs** |  |  |  |  |
| Systolic blood pressure, mmHg | 127 (19) | 127 (20) | 128 (18) | 0.62 |
| Diastolic blood pressure, mmHg | 74 (10) | 74 (10) | 73 (11) | 0.44 |
| HR on echocardiography, BPM | 79 (16) | 79 (14) | 80 (19) | 0.65 |
| Temperature, Celsius | 37.0 (0.7) | 37.0 (0.7) | 37.1 (0.7) | 0.47 |
| Respiratory rate, per min | 18 (16-20) | 18 (16-20) | 18 (17-22) | 0.13 |
| Oxygen saturation, % | 95 (93-97) | 95 (94-97) | 95 (93-97) | 0.95 |
| Oxygen therapy, L/min | 1.0 (0.0-2.5) | 0.0 (0.0-2.0) | 1.0 (0.0-2.5) | 0.34 |
| EWS score, points | 3 (1-5) | 3 (1-4) | 4 (1-5) | 0.07 |
| **Biochemistry** |  |  |  |  |
| Blood pH | 7.47 (0.05) | 7.47 (0.04) | 7.46 (0.05) | 0.50 |
| PCO_2_, kPa | 4.8 (4.2-5.2) | 4.7 (4.3-5.2) | 4.8 (4.1-5.2) | 0.84 |
| Lactate, mmol/L | 1.2 (0.9-1.7) | 1.3 (0.9-1.6) | 1.1 (0.9-1.9) | 0.57 |
| Bicarbonate, mmol/L | 26.1 (3.3) | 26.3 (2.6) | 26.0 (3.8) | 0.63 |
| WBC, x10^9^/L | 7.4 (5.0-9.9) | 7.1 (4.8-9.9) | 7.5 (5.0-9.9) | 0.73 |
| Lymphocytes, x10^9^/L | 1.1 (0.7-1.6) | 1.2 (0.7-1.6) | 1.1 (0.7-1.5) | 0.41 |
| C-reactive protein, mg/L | 58(23-95) | 56 (23-90) | 60 (26-97) | 0.25 |
| Ferritin, µg/L | 637 (322-1250) | 610.0 (336-990) | 637 (302-1280) | 0.22 |
| Procalcitonin, µg/L | 0.2 (0.1-0.6) | 0.2 (0.1-0.4) | 0.2 (0.1-1.3) | 0.047 |
| Creatinine, µmol/L | 76.0 (58.0-103.0) | 68 (56-86) | 95.0 (70-134) | <0.001 |
| Blood urea nitrogen, mmol/L | 6.1 (4.3-10.0) | 5.5 (3.7-7.7) | 7.3 (4.7-11.6) | <0.001 |
| Hemoglobin, mmol/L | 7.3 (1.1) | 7.5 (1.1) | 7.0 (1.1) | 0.006 |
| D-dimer, mg/L (FEU) | 1.3 (0.7-2.3) | 1.3 (0.8-2.6) | 1.2 (0.7-2.1) | 0.93 |
| NT-proBNP, ng/L | 489 (201-1522) | 238 (111-732) | 1192 (389-1789) | <0.001 |
| TnI, ng/L | 13.1 (7.7-36.0) | 11.0 (6.5-19.0) | 142.5 (100.0-440.0) | <0.001 |
| TnT, ng/L | 22.0 (13.0-36.0) | 9.0 (4.0-13.0) | 29.5 (20.0-50.0) | <0.001 |
| **Echocardiography** |  |  |  |  |
| LVEF, % | 59 (53-63) | 61 (54-64) | 57 (50-62) | 0.012 |
| LVMi, g/m^2^ | 86.6 (27.9) | 84.2 (28.0) | 89.7 (27.7) | 0.28 |
| GLS, % | -15.5 (4.2) | -16.3 (3.8) | -14.5 (4.5) | 0.006 |
| LAVi, mL/m^2^ | 19.9 (15.0-27.8) | 20.7 (15.3-25.1) | 19.3 (15.0-30.4) | 0.77 |
| LAEF, % | 46.7 (14.2) | 50.1 (11.3) | 42.6 (16.3) | 0.007 |
| E/A | 1.0 (0.8-1.2) | 1.0 (0.9-1.2) | 1.0 (0.7-1.4) | 0.58 |
| E/e’ (average) | 8.6 (6.8-11.8) | 7.8 (6.6-9.7) | 10.0 (7.9-12.1) | 0.003 |
| TAPSE, cm | 2.1 (0.5) | 2.2 (0.5) | 2.0 (0.5) | 0.014 |
| TR gradient, mmHg ** | 23.2 (9.1) | 22.2 (10.0) | 24.0 (8.2) | 0.29 |
| RV free wall longitudinal strain, % | -19.8 (-24.7--15.9) | -18.9 (-24.2--15.6) | -20.2 (-27.3--15.9) | 0.27 |
| **LUS findings** |  |  |  |  |
| Total number of B-lines | 13 (7-19) | 13 (7-19) | 13 (8-19) | 0.83 |
| LUS score | 2 (1-5) | 2 (1-5) | 2 (1-5) | 0.95 |
| Interstitial syndrome | 34 (19%) | 21 (22%) | 13 (16%) | 0.32 |

Continuous variables are presented as mean (SD) or median (IQR). Proportions are presented as n (%)

BMI=body mass index, BPM=beats per minute, COPD=chronic obstructive pulmonary disease, cTnI=cardiac troponin I, cTnT=cardiac troponin T, E/A=early diastolic mitral inflow relative to late diastolic mitral inflow, E/e'=early diastolic mitral inflow relative to cardiac tissue velocity, EWS=early warning score, GLS=global longitudinal strain, kPa=kilopascals, LAVi=left atrial volume index, LVEF=left ventricular ejection fraction, LVMi=left ventricular mass index, LUS=lung ultrasound, NT-proBNP=N-terminal pro B-type natriuretic peptide, PCO2=partial pressure of carbon dioxide, RV=right ventricular, TAPSE=tricuspid annular plane systolic excursion, TR=tricuspid regurgitation, WBC=white blood cells,

*Sarcoidosis, lung fibrosis, etc.

**In the 164 patients with a measurable peak tricuspid regurgitation continuous wave Doppler signal.

**Supplemental table S3. Demographic and clinical variables according to the median number of B-lines in patients with known heart failure at baseline (n=30).**

|  | **Total** | **14 B-lines** | **>14 B-lines** | **P** |
| --- | --- | --- | --- | --- |
|  | **N=30** | **N=16** | **N=14** |  |
| **Clinical characteristics** |  |  |  |  |
| Age, years | 78 (10) | 74 (12) | 82 (6) | 0.042 |
| Male, n (%) | 19 (63%) | 8 (50%) | 11 (79%) | 0.14 |
| BMI, kg/m2 | 26 (23-32) | 25 (21-31) | 28 (26-34) | 0.097 |
| Hypertension, n (%) | 23 (77%) | 13 (81%) | 10 (71%) | 0.67 |
| Previous ischemic heart disease, n (%) | 13 (45%) | 7 (47%) | 6 (43%) | 1.00 |
| NT-proBNP, ng/L | 1408 (592-3387) | 1412 (747-3679) | 1404 (489-2970) | 0.31 |
| Elevated cardiac troponin, n (%)* | 18 (86%) | 9 (82%) | 9 (90%) | 1.00 |
| **Echocardiography** |  |  |  |  |
| LVEF, % | 44 (35-54) | 48 (30-54) | 43 (35-56) | 1.00 |
| GLS, % | -10.4 (4.8) | -10.1 (4.4) | -10.9 (5.3) | 0.69 |
| E/e' (average) | 9 (7-12) | 9 (7-12) | 9 (8-21) | 0.69 |

Continuous variables are presented as mean (SD) or median (IQR). Proportions are presented as n (%)

BMI=body mass index, BPM=beats per minute, E/e'=early diastolic mitral inflow relative to cardiac tissue velocity, GLS=global longitudinal strain, LAVi=left atrial volume index, LVEF=left ventricular ejection fraction, NT-proBNP=N-terminal pro B-type natriuretic peptide

*Cardiac troponin I and cardiac troponin T above their cut-off for the 99^th^ percentile according to respective center

**Supplemental table S4. Clinical, echocardiographic and lung ultrasound findings according to median CRP of 55) (n=261)**

|  | **Total** | **CRP ≤ median (55)** | **CRP > median (55)** | **P** |
| --- | --- | --- | --- | --- |
|  | N=261 | N=131 | N=130 |  |
| **Clincial characteristics** |  |  |  |  |
| Age, years | 69 (14) | 67 (15) | 71 (13) | 0.017 |
| Male, n (%) | 152 (58%) | 64 (49%) | 88 (68%) | 0.002 |
| BMI, kg/m2 | 25 (23-29) | 25 (23-29) | 26 (23-28) | 0.97 |
| Active smoking, n (%) | 16 (7%) | 6 (5%) | 10 (8%) | 0.14 |
| Hypertension, n (%) | 132 (51%) | 70 (53%) | 62 (48%) | 0.35 |
| Diabetes, n (%) | 59 (23%) | 29 (22%) | 30 (23%) | 0.86 |
| Prevalent heart failure, n (%) | 30 (11%) | 15 (11%) | 15 (12%) | 0.98 |
| Previous ischemic heart disease, n (%) | 28 (11%) | 16 (12%) | 12 (9%) | 0.42 |
| Atrial arrythmia, n (%) | 54 (21%) | 27 (21%) | 27 (21%) | 0.97 |
| COPD, n (%) | 42 (16%) | 24 (18%) | 18 (14%) | 0.33 |
| Asthma, n (%) | 34 (13%) | 17 (13%) | 17 (13%) | 0.96 |
| Other lung disease*, n (%) | 16 (6%) | 10 (8%) | 6 (5%) | 0.32 |
| **Vital signs** |  |  |  |  |
| Systolic blood pressure, (mmHg) | 127 (19) | 128 (19) | 125 (18) | 0.24 |
| HR, (BPM) | 81 (17) | 80 (17) | 81 (17) | 0.72 |
| Temperature, (Degrees Celsius) | 37.0 (0.7) | 36.9 (0.6) | 37.2 (0.7) | 0.003 |
| Respiratory rate, per min | 18 (17-20) | 18 (16-20) | 20 (18-20) | 0.013 |
| Oxygen saturation, % | 95 (93-96) | 95 (94-97) | 95 (93-96) | 0.09 |
| Oxygen therapy, L/min | 1.0 (0.0-3.0) | 0.0 (0.0-2.0) | 1.2 (0.0-4.0) | <0.001 |
| EWS score, points | 3 (1-5) | 2 (1-4) | 4 (2-5) | <0.001 |
| **Biochemistry** |  |  |  |  |
| WBC, ng/L | 7.1 (5.0-9.9) | 7.2 (5.0-9.9) | 6.8 (5.0-9.8) | 0.74 |
| Lymphocytes, ng/L | 1.1 (0.7-1.5) | 1.3 (0.9-1.8) | 0.9 (0.6-1.3) | <0.001 |
| C-reactive protein, mg/L | 55 (23-93) | 23 (11-36) | 93 (68-139) | <0.001 |
| Ferritin, µg/L | 609 (304-1250) | 460 (222-840) | 852 (457-1530) | <0.001 |
| Procalcitonin, µg/L | 0.2 (0.1-0.5) | 0.1 (0.1-0.2) | 0.2 (0.1-0.9) | 0.009 |
| Creatinine, µmol/L | 75 (58-99) | 70(57-90) | 82 (62-109) | 0.009 |
| Blood urea nitrogen, mmol/L | 5.8 (4.0-9.5) | 5.6 (4.0-8.6) | 6.0 (4.0-9.8) | 0.28 |
| Hemoglobin, mmol/L | 7.4 (1.1) | 7.4 (1.1) | 7.4 (1.2) | 0.59 |
| D-dimer, mg/L (FEU) | 1.3 (0.8-2.4) | 1.1 (0.6-2.1) | 1.6 (0.9-2.5) | 0.029 |
| NT-proBNP, ng/L | 409 (178-1582) | 390 (150-1247) | 444 (203-2013) | 0.09 |
| cTnI, ng/L | 14 (8-36) | 9 (6-17) | 18 (11-54) | 0.002 |
| cTnT, ng/L | 22 (13-36) | 22 (17-33) | 23.5 (13-54) | 0.61 |
| Elevated troponins, n (%) ** | 82 (46%) | 36 (43%) | 46 (49%) | 0.46 |
| **Echocardiography** |  |  |  |  |
| LVEF, % | 59 (54-63) | 59 (54-63) | 59 (53-63) | 0.94 |
| LVMi, g/m2 | 84.4 (26.8) | 85.4 (26.8) | 83.4 (26.8) | 0.62 |
| GLS, % | -15.8 (4.4) | -16.0 (4.1) | -15.6 (4.7) | 0.53 |
| LAVi, mL/m2 | 20.1 (15.1-26.1) | 19.5 (14.8-25.4) | 20.5 (15.5-28.0) | 0.33 |
| E/A | 1.0 (0.8-1.2) | 1.0 (0.8-1.2) | 1.0 (0.9-1.3) | 0.25 |
| E/e' (average) | 8.4 (6.7-11.8) | 8.6 (6.7-11.2) | 8.4 (6.8-12.0) | 0.87 |
| TAPSE, cm | 2.0 (0.5) | 2.0 (0.4) | 2.0 (0.5) | 0.53 |
| Peak TR gradient, mmHg | 22.4 (9.6) | 21.2 (10.0) | 23.5 (9.1) | 0.14 |
| RV free wall longitudinal strain, % | -18.7 (-24.4 –  -15.6) | -18.6 (-24.7 –  -15.1) | -18.8 (-24.3 –  -15.9) | 0.91 |
| **LUS findings** |  |  |  |  |
| Total number of B-lines | 13 (8-19) | 12 (7-17) | 16 (9-22) | 0.001 |
| LUS score | 2 (1-5) | 2 (0-4) | 3 (1-6) | 0.006 |
| Interstitial syndrome, n (%) | 49 (19%) | 21 (16%) | 28 (22%) | 0.25 |

Continuous variables are presented as mean (SD) or median (IQR). Proportions are presented as n (%)

BMI=body mass index, BPM=beats per minute, COPD=chronic obstructive pulmonary disease, cTnI=cardiac troponin I, cTnT=cardiac troponin T, E/A=early diastolic mitral inflow relative to late diastolic mitral inflow, E/e'=early diastolic mitral inflow relative to cardiac tissue velocity, EWS=early warning score, GLS=global longitudinal strain,

LAVi=left atrial volume index, LVEF=left ventricular ejection fraction, LVMi=left ventricular mass index, LUS=lung ultrasound, NT-proBNP=N-terminal pro B-type natriuretic peptide,

PCO2=partial pressure of carbon dioxide, RV=right ventricular, TAPSE=tricuspid annular plane systolic excursion, TR=tricuspid regurgitation, WBC=white blood cells

*Sarcoidosis, lung fibrosis, etc.

**Cardiac troponin I and cardiac troponin T above their cut-off for the 99^th^ percentile according to the respective centers
